# Rheumatoid arthritis and stroke risk: a systematic review and meta-analysis

**DOI:** 10.7717/peerj.20568

**Published:** 2026-01-29

**Authors:** Lulu Yang, Xinyu Liu, Guangyuan Yang, Hongmei Wu, Xingsen Li, Jinyu Xuan, Shuxin Dong

**Affiliations:** 1 Rehabilitation Medical College, Jiamusi University, Jiamusi, Heilongjiang, China; 2 Department of Rehabilitation Medicine, The First Affiliated Hospital of Jiamusi University, Jiamusi, Heilongjiang, China; 3 The Third Affiliated Hospital, Jiamusi University, Jiamusi, Heilongjiang, China; 4 Heilongjiang Provincial Key Laboratory of Pediatric Neurological Rehabilitation, Jiamusi, Heilongjiang, China; 5 Cardiovascular Medicine,The First Affiliated Hospital of Jiamusi University, Jiamusi, Heilongjiang, China

**Keywords:** Rheumatoid arthritis, Meta-analysis, Stroke, Systematic review

## Abstract

**Objectives:**

This meta-analysis aims to evaluate the association between rheumatoid arthritis (RA) and stroke risk. Methods: PubMed, Cochrane Library, and Embase were searched for observational studies published from database inception to October 7, 2025, using medical subject headings (MeSH) and keywords. Random effect models were used to calculate odds ratios (OR) and 95% confidence intervals (CI) for evaluating the associations between RA and stroke risk. All statistical analyses were performed using Stata statistical software version 17.0. The funnel plot, Egger’s test and Begg’s test were used to evaluate publication bias.

**Results:**

This meta-analysis included 12 observational studies with a total of 1,715,001 participants, published between 2003 and 2025. The pooled analysis revealed a significant association between RA and increased stroke risk (OR = 1.35; 95% CI [1.26–1.45]; *P* = 0.000). Subgroup analysis showed that women with RA had a slightly higher stroke risk than men (OR = 1.60; 95% CI [1.19–2.16]; *P* = 0.002). Additionally, RA patients aged over 65 were at higher risk of stroke (OR = 1.24; 95% CI [1.02–1.50]; *P* = 0.032). No significant publication bias was detected, and sensitivity analyses confirmed the robustness of our findings.

**Conclusions:**

This meta-analysis demonstrates that RA is associated with an increased risk of stroke, supporting the recognition of RA as an independent stroke risk factor.

## Introduction

Stroke is an acute syndrome and a leading cause of death and disability worldwide (Murphy & Werring, 2020). The Global Burden of Disease (GBD) study reported 11.9 million incident strokes, 93.8 million prevalent strokes, 7.25 million stroke-related deaths, and 160 million Disability-Adjusted Life Years (DALYs) lost to stroke in 2021 (Diseases & Injuries, 2024). Early identification of stroke risk factors is crucial for prevention. Several quantitative meta-analyses have identified key risk factors for stroke, including dementia, sleep insufficiency, migraines, dietary protein intake, cold spell and abnormal body weight (Fan et al., 2023; Kuźma et al., 2018; Li et al., 2016; Zhang et al., 2021; Zhang et al., 2016; Zhao et al., 2024). Recently, rheumatoid arthritis (RA) has emerged as a potential risk factor for stroke due to its association with persistent synovial inflammation and progressive joint destruction (Agca et al., 2017). This chronic inflammation stimulates the release of enzymes, pro-inflammatory mediators, and cytokines, resulting in synovial tissue degradation (Miller, Miller & Malfait, 2014). The increased production of inflammatory cytokines in the joints is a key mechanism connecting rheumatoid arthritis to a higher risk of stroke. These cytokines can enter the bloodstream and increase the production of adhesion molecules and other pro-inflammatory molecules. This leads to monocyte and leukocyte adhesion to the endothelial cells of blood vessels, migration into the vessel walls, and eventually triggering strokes (Libby, 2009; Zaman et al., 2000).

RA is a chronic autoimmune inflammatory disease associated with disability and premature mortality. In 2020, an estimated 17.6 million people worldwide were living with RA, with a global age-standardized prevalence of 208.8 cases per 100,000 population, reflecting a 14.1% increase since 1990 (GBD 2021 Rheumatoid Arthritis Collaborators, 2023). Current evidence suggests RA may be linked to an increased risk of stroke (Nadareishvili et al., 2008). However, the role of RA in stroke is still controversial. A previous meta-analysis showed that RA are associated with increased cardiovascular risk, likely due to the limited number of studies included (only four case-control studies). Simultaneously, they did not analyze the influence of age, sex, country, stroke type, and study design (Lévy et al., 2008). Therefore, this systematic review and meta-analysis aim to evaluate the influence of RA on stroke risk globally.

## Methods

This meta-analysis was conducted in accordance with the Preferred Reporting Items for Systematic Reviews and Meta-Analyses (PRISMA) guidelines (Page et al., 2021). The protocol was preregistered on the International Prospective Register of Systematic Reviews (PROSPERO) platform (registration number: CRD42024621518).

### Data sources and searches

PubMed, Cochrane, and Embase were searched for observational studies published from database inception through October 7, 2025, with no language restrictions. The search strategy combined medical subject headings (MeSH) and keywords, including “Stroke”, “Cerebrovascular Apoplexy”, “Brain Vascular Accident”, “Cerebrovascular Accident”, and “Rheumatoid Arthritis”. The full PubMed search strategy is presented in Table S1 We also reviewed the reference lists of included studies and relevant meta-analyses to identify additional trials.

### Eligibility driteria

We included cohort or cross-sectional studies that examined the association between RA and stroke risk, with clear diagnoses of both conditions.

Studies were excluded if they did not report odds ratios (OR) with corresponding 95% confidence intervals (CI). In cases where multiple studies reported data from the same cohort, we selected the study with the longest follow-up or largest sample size. Conference abstracts, study protocols, and duplicate publications were also excluded.

### Study selection

Two reviewers (YLL and LXY) independently screened studies for eligibility based on the inclusion and exclusion criteria. Initially, duplicate and irrelevant articles were removed based on titles and abstracts. Full texts of potentially eligible studies were then reviewed to confirm eligibility. Disagreements were resolved through discussion with a third reviewer (WHM).

### Data extraction

Data extraction was performed independently by the two primary reviewers (YLL and LXY),following established guidelines for systematic reviews and meta-analyses (Taylor, Mahtani & Aronson, 2021). Data extracted included: first author, publication year, study type, sample size, follow-up duration, participant age and sex, RA and stroke diagnoses, and adjusted confounders. Any discrepancies were resolved by consensus with (WHM).

### Risk of bias assessment

Study quality was assessed using the Newcastle-Ottawa Scale (NOS) for cohort studies (Wells et al., 2000), with ratings ranging from 0 to 9 stars. Four stars were allocated for participant selection and exposure measurement, two for comparability, and three for outcome assessment and follow-up adequacy. Studies were classified as low (0–3 stars), moderate (4–6 stars), or high (7–9 stars) quality.

For cross-sectional studies, the American Agency for Health Care Quality and Research (AHRQ) (Rostom et al., 2004) quality evaluation criteria were applied. Each item was scored as ‘1’ for a ‘YES’ response, or ‘0’ for ‘UNCLEAR’ or ‘NO’. The total scores were grouped into low (0–3), moderate (4–7), and high (8–11) quality.

### Statistical analysis

The adjusted OR and 95% CI from each study were used to estimate the association between RA and stroke risk. Heterogeneity was assessed using the chi-square test and I^2^ statistics. Due to differences in the population characteristics and RA duration, there was significant clinical heterogeneity in the included studies. So, regardless of statistical heterogeneity, we would use a random effects model to analyze the data. Sensitivity analyses were conducted by sequentially excluding each study to test the robustness of the results. Publication bias was evaluated visually using funnel plots and statistically with Egger’s test and Begg’s test. We performed subgroup analyses by gender and study type. All statistical analyses were performed using Stata version 17.0 (StataCorp, College Station, TX, USA).

## Results

### Literature search

**Figure 1:**
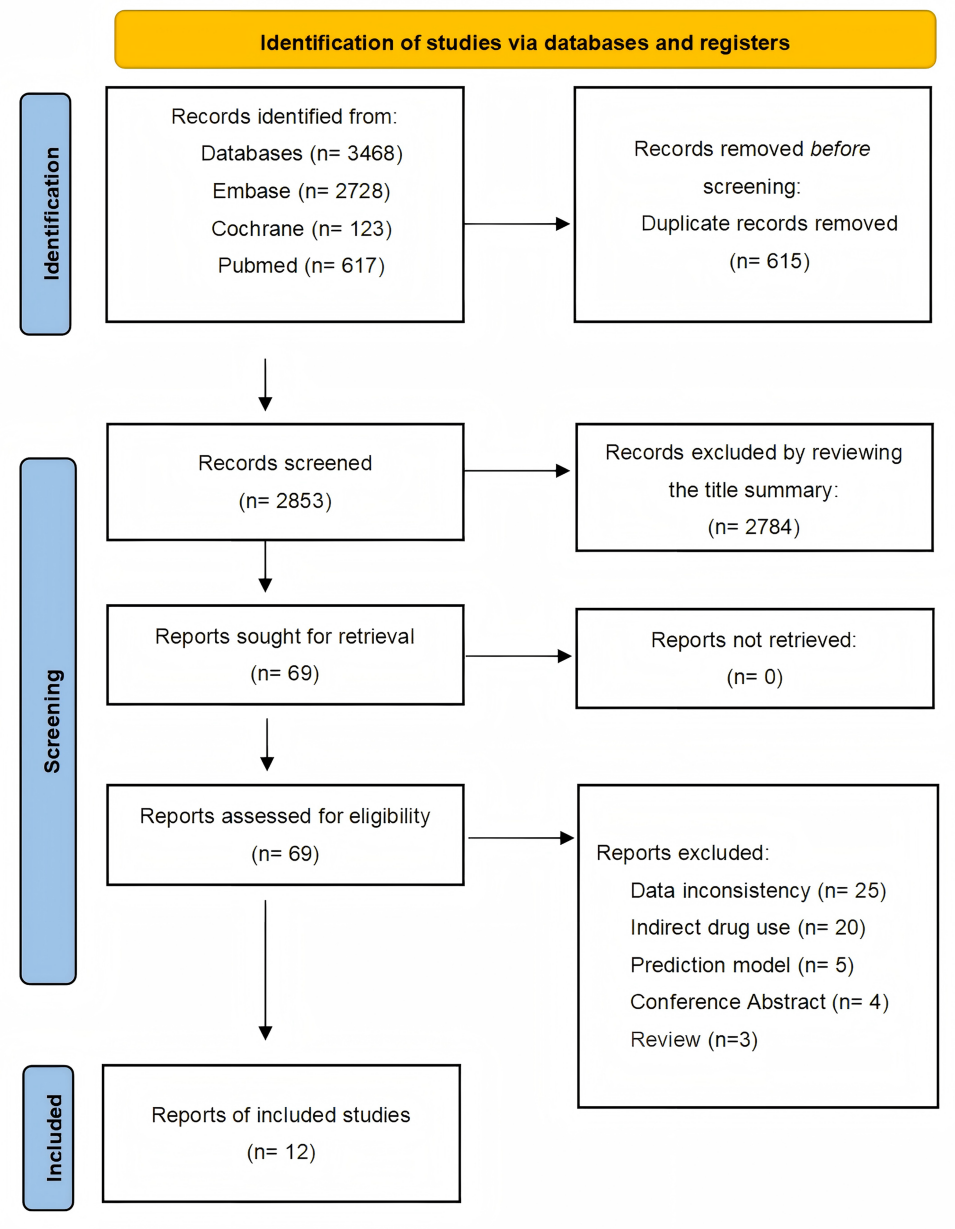
Studies screening process.

A systematic search of observational studies published before October 7, 2025, identified 3,468 results. After screening titles and abstracts, 15 articles were considered potentially relevant, and 12 studies (Baviera et al., 2021; Chen et al., 2018a; Chen et al., 2018b; Wolfe, Breundlich & Straus, 2003; Kang et al., 2022; Lee et al., 2021; Liou et al., 2014; Logstrup et al., 2021; Shin et al., 2025; Tiosano et al., 2017; Trommer et al., 2021; Zou et al., 2017) were ultimately included. The selection process is detailed in Fig. 1.

### Study characteristics

This meta-analysis includes 12 studies (Baviera et al., 2021; Chen et al., 2018a; Chen et al., 2018b; Wolfe, Breundlich & Straus, 2003; Kang et al., 2022; Lee et al., 2021; Liou et al., 2014; Logstrup et al., 2021; Shin et al., 2025; Tiosano et al., 2017; Trommer et al., 2021; Zou et al., 2017) with a total sample size of 1,715,001 individuals, ranging from 2,044 to 818,814 participants. These studies, published between 2003 and 2025, comprised 10 cohort studies (Baviera et al., 2021; Chen et al., 2018a; Chen et al., 2018b; Wolfe, Breundlich & Straus, 2003; Kang et al., 2022; Lee et al., 2021; Liou et al., 2014; Logstrup et al., 2021; Shin et al., 2025; Trommer et al., 2021) and 2 cross-sectional studies (Tiosano et al., 2017; Zou et al., 2017). Eight studies were conducted in developed countries (USA, Germany, Italy, Denmark, Israel, Korea) and four in developing countries (China, Taiwan). Age, sex, and comorbidities were the most common potential confounders in the adjusted models. Key study characteristics are presented in Table 1.

**Table 1:** Basic characteristics of the included studies.

**Author**	**Year**	**Country**	**Study type**	**Sample size**	**Follow-up years**	**Age (Mean ± SD)**	**Sex**	**Diagnosis of RA/stroke**	**Confounders adjusted**	**NOS scores**
Wolfe, Breundlich & Straus (2003)	2003	USA	Retrospective cohort	Total: 11,572	6 months	59.8 ± 13.0 66.0 ± 11.22	Male 9,093 (23.1%) RA Male 2,479 (18.3%) OA	ICD-10	Age, sex, education level, smoking, income, hypertension, and body mass index.	8
Lee et al. (2021)	2021	Korea	Retrospective cohort	Total: 16,590 Stroke: 64	12 averages	54 ± 8.7	Male 735 (26.6%) RA Male 3,675 (26.6%) Control	ICD-10	Age, sex, and comorbidities, including hypertension, diabetes mellitus, and dyslipidemia.	9
Kang et al. (2022)	2022	Korea	Retrospective cohort	Total: 818,814 Stroke: 1,830	8 averages	54.6 ± 11.6	Male 36,075 (26.4%) RA Male 180,375 (26.4%) Controls Female 100,394 (73.6%) RA Female 501,970 (73.6%) Controls	ICD-10-CM	Age, sex, smoking, alcohol drinking, regular exercise, obesity, DM, hyperlipidemia and income.	7
Trommer et al. (2021)	2021	Germany	Retrospective cohort	Total: 58,212 Stroke: 842	16 averages	54.8 ± 14.4	Male 29,106 (35.0%) Female 29,106 (65.0%)	ICD-10	Age, sex.	8
Logstrup et al. (2021)	2020	Denmark	Retrospective cohort	Total: 90,192	22 averages	NA	Female 10,037 (66.8%) RA Female 50,185 (66.8%) Controls	ICD-10	Age, sex and co-morbidity.	8
Baviera et al. (2021)	2021	Italy	Retrospective cohort	Total: 516,047 Stroke: 10,476	13 averages	59 ± 14.3 RA 45.3 ± 19.1 No RA	Female 11,690 (72.8%) RA Female 255,803 (51.1%) Non-RA	ICD-9-CM/ ICD-10	Age, sex, index year and comorbidities.	8
Chen et al. (2018a)	2018	Taiwan	Retrospective cohort	Total: 52,840 Stroke: 114	6 averages	NA	Male 2,742 (25.95%) RA Male 10,968 (25.95%) Non-RA	ICD-9-CM	Age, sex, hypertension, diabetes mellitus, hyperlipidemia, cancer, mild liver disease, COPD, glucocorticoids, and biologics.	7
Zou et al. (2017)	2017	China	Cross-sectional study	Total: 2,044 Stroke: 56	11 averages	58.5 ± 0.4 Controls 57.8 ± 0.4 RA	Female 702 (68.7%)	ICD-10	NA	7
Tiosano et al. (2017)	2017	Israel	Cross-sectional study	Total: 69,755 Stroke: 4,361	NA	≤65y: 49.8 ± 13.2 >65y: 75.8 ± 7.09	≤65y: Female 5,115 (76.7%)RA 25,454 (76.7%) No RA >65y: Female 3,988 (77.9%) RA 19,135 (77.3%) No RA	ICD-10	NA	8
Liou et al. (2014)	2014	Taiwan	Retrospective cohort	Total: 30,570 Stroke: 383	4 averages	NA	Male 1,752 (28.7%) RA 7,008 (28.7%) Control Female 4,362 (71.3%) RA 17448 (71.3%) Control	ICD-9-CM	Age, gender, urbanization level, hyperlipidemia, coronary heart diseases, congestive heart failure, renal disease, atrial fibrillation, valvular heart disease.	7
Chen et al. (2018b)	2018	Taiwan	Retrospective cohort	Total: 3,190 Stroke: 521	6 averages	71.42 ± 10.93 RA 71.34 ± 10.96 No RA	Male 300 (47.02%) RA 1,200 (47.02%) Control	ICD-9-CM	Age, sex, urbanicity, NIHSS, chronic kidney disease.	8
Shin et al. (2025)	2025	Korea	Retrospective cohort	Total: 45,175 Stroke: 3950	5 averages	57.4 ± 9.6	Male 43,956 (24.3%)	ICD-10	Sex, age, income, smoking, alcohol drinking, physical activity, and obesity, hyperlipidemia, chronic kidney disease, and atrial fibrillation.	8

### Quality assessment

We assessed the quality of all 12 studies using the NOS and AHRQ scales, with scores summarized in Table 1. The average NOS score for cohort studies was 7.75, with each cohort study scoring 7 or higher, indicating high quality. Cross-sectional studies received a score of 8, reflecting high quality according to the AHRQ criteria.

### Rheumatoid arthritis and risk of stroke

**Figure 2:**
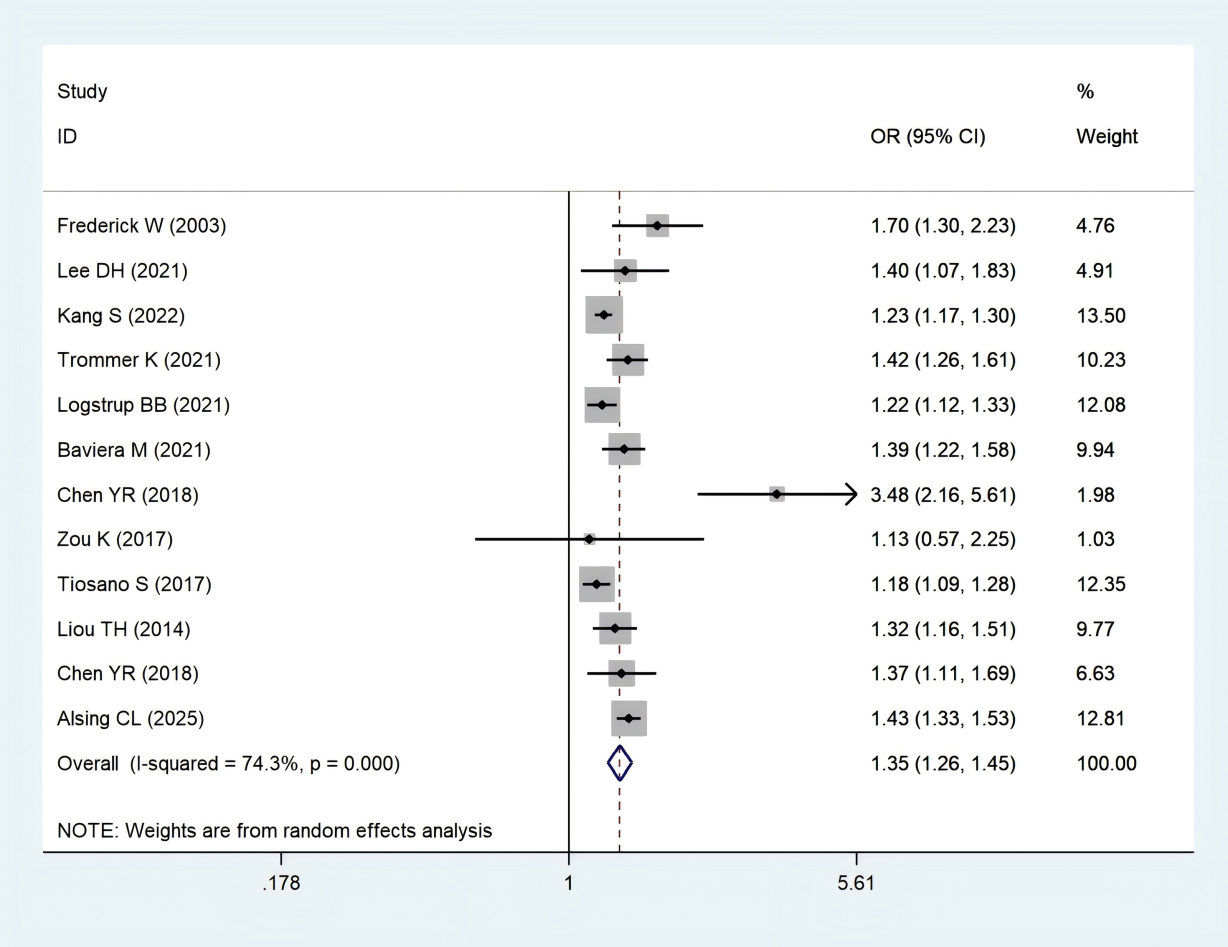
Meta-analysis of the risk of stroke caused by RA. Studies: Baviera et al. (2021); Chen et al. (2018a); Chen et al. (2018b); Wolfe, Breundlich & Straus (2003); Kang et al. (2022); Lee et al. (2021); Liou et al. (2014); Logstrup et al. (2021); Shin et al. (2025); Tiosano et al. (2017); Trommer et al. (2021); Zou et al. (2017).

Twelve studies (Baviera et al., 2021; Chen et al., 2018a; Chen et al., 2018b; Wolfe, Breundlich & Straus, 2003; Kang et al., 2022; Lee et al., 2021; Liou et al., 2014; Logstrup et al., 2021; Shin et al., 2025; Tiosano et al., 2017; Trommer et al., 2021; Zou et al., 2017) examined the relationship between rheumatoid arthritis (RA) and stroke risk. The pooled odds ratio (OR) was 1.35 (95% CI [1.26–1.45]; *P* = 0.000) (Fig. 2). Sensitivity analysis confirmed the robustness of these results, as none of the individual studies reversed the pooled effect size (Fig. S1).

**Table 2:** Subgroup analysis for the risk of stroke in patients with RA.

Subgroups	Included studies	OR (95% CI)	Heterogeneity		*P* value of pooled effect
			I^2^ (%)	*P*-values	
Sex					
Female	5	1.60 (1.19, 2.16)	92.8%	0.000	0.002
Male	5	1.44 (1.08, 1.94)	85.6%	0.000	0.015
Study type					
Retrospective cohort	10	1.38 (1.28, 1.50)	75.3%	0.000	0.000
Cross-sectional	2	1.18 (1.09, 1.28)	0.0%	0.902	0.000
Age					
≤65	2	1.56 (1.21, 2.03)	53.8%	0.141	0.001
>65	3	1 .24 (1.02, 1.50)	73.7%	0.022	0.032
Stroke type					
Ischemic stroke	3	1.75 (1.27, 2.39)	84.0%	0.002	0.001
Hemorrhagic stroke	3	1.30 (1.14, 1.48)	0.0%	0.407	0.000
Country					
Developed country	8	1.32 (1.23,1.42)	72.7%	0.001	0.000
Developing country	4	1.59 (1.15,2.20)	72.7%	0.002	0.005

### Subgroup analysis

Subgroup analyses by age, sex, and study design showed no significant difference in the association between RA and stroke risk based on study type. However, women with RA appeared to have a slightly higher stroke risk compared to men.

The pooled adjusted odds ratio for ischemic stroke in RA patients was 1.75 (95% CI [1.27–2.39]; *P* = 0.001). In patients aged over 65 years, the stroke risk was estimated at 1.24 (95% CI [1.02–1.50]; *P* = 0.032). (Table 2).

### Publications bias

**Figure 3:**
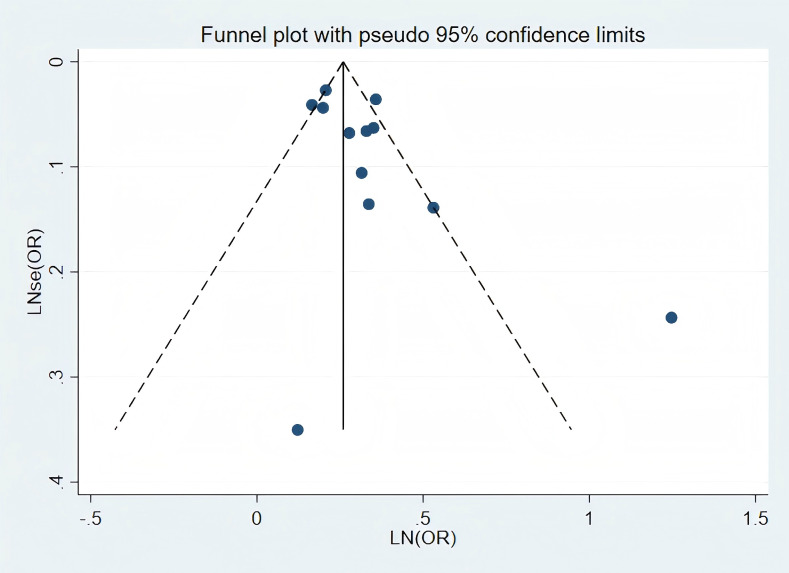
Publication bias of the risk of stroke caused by RA.

Visual inspection of the funnel plot revealed no significant publication bias in the association between RA and stroke risk (Fig. 3). Similarly, Publication bias was formally assessed using Begg’ s test (*P* = 0.244; Fig. S2) and Egger’s test (*P* = 0.067; Fig. S3), which was corrected using the trim-and-fill method (Fig. S4).

## Discussion

### Main findings

This meta-analysis included 12 studies encompassing a total of 1,715,001 individuals, providing a comprehensive evaluation of the association between RA and stroke. We observed a significant increase in stroke risk among individuals with RA, with a 1.35-fold higher risk compared to non-RA controls.

### Interpretation of findings

The prevalence of both RA and stroke continues to rise, with cardiovascular disease being a leading cause of mortality in individuals with RA. Therefore, our study aimed to further investigate this association, offering more robust evidence based on a larger number of cohort and cross-sectional studies. Additionally, we conducted a subgroup analysis to assess the impact of RA on different types of stroke, which contributes to a deeper understanding of their specific effects. Our findings emphasize the importance of recognizing RA as a significant risk factor for stroke, supporting the need for tailored healthcare strategies for individuals with RA.

An alternative investigation examined the study on cardiovascular risk factors in a population with rheumatoid arthritis (McEntegart et al., 2001; Semb et al., 2010). However, the lack of an identified association between RA and stroke risk in their analysis was likely due to an underpowered study. In contrast, our study reanalyzed the relationship between RA and stroke, revealing a clear increase in stroke risk in this population.

The precise mechanism linking RA and stroke remains under ongoing investigation. RA is a systemic autoimmune disorder characterized by chronic inflammation of the smaller synovial joints, which causes pain and deformities (Behrouz, 2014). Chronic inflammation in RA accelerates atherosclerosis *via* endothelial dysfunction, infiltration of inflammatory cells in plaques, and acute plaque rupture, ultimately contributing to stroke risk (Hansson, 2005; Sattar et al., 2003). Moreover, RA can induce necrotizing vasculitis in small- and medium-sized vessels, particularly affecting cerebral vessels (Makol, Matteson & Warrington, 2015). Small-vessel vasculitis is commonly diagnosed through clinical suspicion, MRI evidence of parenchymal or meningeal pathology, and inflammation markers in the cerebrospinal fluid (Makol, Matteson & Warrington, 2015).

RA also has important cardiovascular implications. Several studies have shown an increased prevalence of atrial fibrillation (AF) in RA patients (Engelmann & Svendsen, 2005; Guo, Lip & Apostolakis, 2012; Ungprasert, Srivali & Kittanamongkolchai, 2017), with multifactorial mechanisms that increase the risk of clot formation and, consequently, stroke (Lindhardsen et al., 2012). Furthermore, chronic inflammation in RA patients raises the risk of valvular nodules and thickening, which are associated with a higher risk of stroke (Corrao et al., 2013).

In autoimmune diseases like RA, the occurrence of cardiovascular events is influenced by both traditional cardiovascular risk factors, such as smoking, dyslipidemia, diabetes, and hypertension, as well as by the disease’s systemic effects (Anyfanti et al., 2024). Additionally, some studies suggest that genetic susceptibility may further elevate cardiovascular risk in RA patients (Farragher et al., 2008; Van den Oever, Van Sijl & Nurmohamed, 2013; Yuan et al., 2022).

Although the association between RA and stroke is well-established, the precise mechanisms underlying this relationship warrant further investigation. A more comprehensive understanding of these mechanisms is crucial for mitigating stroke risk and improving outcomes for RA patients who experience stroke.

### Implications and limitations

Our meta-analysis synthesizes existing evidence regarding the link between RA and stroke risk. It underscores the importance of closely monitoring stroke risk in RA patients, which may lead to early identification of high-risk individuals and more proactive interventions. One of the strengths of our study is that it is the first systematic review to consolidate the current best evidence on this association. However, there are certain limitations. First, the majority of the included studies were retrospective, which can introduce inherent biases. Additionally, our meta-analysis did not include covariate analysis, and the adjustment factors used in the studies varied. Some studies did not apply any adjustments, which could influence the precision of the results. Despite these variations, most studies included in our review accounted for confounding factors, thereby reducing bias and ensuring the reliability of our findings. This strengthens the clinical applicability of our conclusions.

## Conclusions

This meta-analysis indicates that RA is associated with an increased risk of stroke. However, a deeper understanding of the underlying pathophysiology of this association is necessary.

## Supplemental Information

10.7717/peerj.20568/supp-1 Supplemental Information 1 PRISMA checklist

10.7717/peerj.20568/supp-2 Supplemental Information 2 Original data

10.7717/peerj.20568/supp-3 Supplemental Information 3 The retrieval strategies and retrieval results of each database.

## Additional Information and Declarations

### Data Availability

The following information was supplied regarding data availability: The raw data are available in the Supplemental File.

## References

[ref-1] Agca R, Heslinga S, Rollefstad S, Heslinga M, McInnes I, Peters M, Kvien T, Dougados M, Radner H, Atzeni F (2017). EULAR recommendations for cardiovascular disease risk management in patients with rheumatoid arthritis and other forms of inflammatory joint disorders: 2015/2016 update. Annals of the Rheumatic Diseases.

[ref-2] Anyfanti P, Ainatzoglou A, Angeloudi E, Michailou O, Defteraiou K, Bekiari E, Kitas GD, Dimitroulas T (2024). Cardiovascular risk in rheumatoid arthritis: considerations on assessment and management. Mediterranean Journal of Rheumatology.

[ref-3] Baviera M, Cioffi G, Colacioppo P, Tettamanti M, Fortino I, Roncaglioni MC (2021). Temporal trends from 2005 to 2018 in deaths and cardiovascular events in subjects with newly diagnosed rheumatoid arthritis. Internal and Emergency Medicine.

[ref-4] Behrouz R (2014). The risk of ischemic stroke in major rheumatic disorders. Neuroimmunology.

[ref-5] Chen YR, Hsieh FI, Chang CC, Chi NF, Wu HC, Chiou HY (2018a). The effect of rheumatoid arthritis on the risk of cerebrovascular disease and coronary artery disease in young adults. Journal of the Chinese Medical Association.

[ref-6] Chen YR, Hsieh FI, Lien LM, Hu CJ, Jeng JS, Peng GS, Tang SC, Chi NF, Sung YF, Chiou HY (2018b). Rheumatoid arthritis significantly increased recurrence risk after ischemic stroke/transient ischemic attack. Journal of Neurology.

[ref-7] Corrao S, Messina S, Pistone G, Calvo L, Scaglione R, Licata G (2013). Heart involvement in rheumatoid arthritis: systematic review and meta-analysis. Cardiology.

[ref-8] Diseases GBD, Injuries C (2024). Global incidence, prevalence, years lived with disability (YLDs), disability-adjusted life-years (DALYs), and healthy life expectancy (HALE) for 371 diseases and injuries in 204 countries and territories and 811 subnational locations, 1990–2021: a systematic analysis for the global burden of disease study 2021. Lancet.

[ref-9] Engelmann MD, Svendsen JH (2005). Inflammation in the genesis and perpetuation of atrial fibrillation. European Heart Journal.

[ref-10] Fan JF, Xiao YC, Feng YF, Niu LY, Tan X, Sun JC, Leng YQ, Li WY, Wang WZ, Wang YK (2023). A systematic review and meta-analysis of cold exposure and cardiovascular disease outcomes. Frontiers in Cardiovascular Medicine.

[ref-11] Farragher TM, Goodson NJ, Naseem H, Silman AJ, Thomson W, Symmons D, Barton A (2008). Association of the HLA–DRB1 gene with premature death, particularly from cardiovascular disease, in patients with rheumatoid arthritis and inflammatory polyarthritis. Arthritis & Rheumatology.

[ref-12] GBD 2021 Rheumatoid Arthritis Collaborators (2023). Global, regional, and national burden of rheumatoid arthritis, 1990–2020, and projections to 2050: a systematic analysis of the global burden of disease study 2021. The Lancet Rheumatology.

[ref-13] Guo Y, Lip GY, Apostolakis S (2012). Inflammation in atrial fibrillation. JACC Journals.

[ref-14] Hansson GK (2005). Inflammation, atherosclerosis, and coronary artery disease. New England Journal of Medicine.

[ref-15] Kang S, Han K, Jung JH, Eun Y, Kim IY, Hwang J, Koh EM, Lee S, Cha HS, Kim H, Lee J (2022). Associations between cardiovascular outcomes and rheumatoid arthritis: a nationwide population-based cohort study. Journal of Clinical Medicine.

[ref-16] Kuźma E, Lourida I, Moore SF, Levine DA, Ukoumunne OC, Llewellyn DJ (2018). Stroke and dementia risk: a systematic review and meta-analysis. Alzheimer’s & Dementia.

[ref-17] Lee DH, Sheen SH, Lee DG, Jang JW, Lee DC, Shin SH, Han IB, Hong JB, Kim H, Sohn S (2021). Association between ischemic stroke and seropositive rheumatoid arthritis in Korea: a nationwide longitudinal cohort study. PLOS ONE.

[ref-18] Lévy L, Fautrel B, Barnetche T, Schaeverbeke T (2008). Incidence and risk of fatal myocardial infarction and stroke events in rheumatoid arthritis patients. A systematic review of the literature. Clinical and Experimental Rheumatology.

[ref-19] Li W, Wang D, Cao S, Yin X, Gong Y, Gan Y, Zhou Y, Lu Z (2016). Sleep duration and risk of stroke events and stroke mortality: a systematic review and meta-analysis of prospective cohort studies. International Journal of Cardiology.

[ref-20] Libby P (2009). Molecular and cellular mechanisms of the thrombotic complications of atherosclerosis. Journal of Lipid Research.

[ref-21] Lindhardsen J, Ahlehoff O, Gislason GH, Madsen OR, Olesen JB, Svendsen JH, Torp-Pedersen C, Hansen PR (2012). Risk of atrial fibrillation and stroke in rheumatoid arthritis: Danish nationwide cohort study. BMJ.

[ref-22] Liou TH, Huang SW, Lin JW, Chang YS, Wu CW, Lin HW (2014). Risk of stroke in patients with rheumatism: a nationwide longitudinal population-based study. Scientific Reports.

[ref-23] Logstrup BB, Ellingsen T, Pedersen AB, Darvalics B, Olesen KKW, Botker HE, Maeng M (2021). Cardiovascular risk and mortality in rheumatoid arthritis compared with diabetes mellitus and the general population. Rheumatology.

[ref-24] Makol A, Matteson EL, Warrington KJ (2015). Rheumatoid vasculitis: an update. Current Opinion in Rheumatology.

[ref-25] McEntegart A, Capell HA, Creran D, Rumley A, Woodward M, Lowe GD (2001). Cardiovascular risk factors, including thrombotic variables, in a population with rheumatoid arthritis. Rheumatology.

[ref-26] Miller RE, Miller RJ, Malfait AM (2014). Osteoarthritis joint pain: the cytokine connection. Cytokine.

[ref-27] Murphy SJ, Werring DJ (2020). Stroke: causes and clinical features. Medicine.

[ref-28] Nadareishvili Z, Michaud K, Hallenbeck JM, Wolfe F (2008). Cardiovascular, rheumatologic, and pharmacologic predictors of stroke in patients with rheumatoid arthritis: a nested, case–control study. Arthritis Care & Research.

[ref-29] Page MJ, McKenzie JE, Bossuyt PM, Boutron I, Hoffmann TC, Mulrow CD, Shamseer L, Tetzlaff JM, Akl EA, Brennan SE, Chou R, Glanville J, Grimshaw JM, Hrobjartsson A, Lalu MM, Li T, Loder EW, Mayo-Wilson E, McDonald S, McGuinness LA, Stewart LA, Thomas J, Tricco AC, Welch VA, Whiting P, Moher D (2021). The PRISMA 2020 statement: an updated guideline for reporting systematic reviews. BMJ.

[ref-30] Rostom A, Dubé C, Cranney A, Saloojee N, Sy R, Garritty C, Sampson M, Zhang L, Yazdi F, Mamaladze V (2004). Celiac disease: summary.

[ref-31] Sattar N, McCarey DW, Capell H, McInnes IB (2003). Explaining how high-grade systemic inflammation accelerates vascular risk in rheumatoid arthritis. Circulation.

[ref-32] Semb AG, Kvien TK, Aastveit AH, Jungner I, Pedersen TR, Walldius G, Holme I (2010). Lipids, myocardial infarction and ischaemic stroke in patients with rheumatoid arthritis in the Apolipoprotein-related mortality risk (AMORIS) study. Annals of the Rheumatic Diseases.

[ref-33] Shin A, Kang S, Jung JH, Cho IY, Han KD, Kim S, Kim SY, Shin DW, Kim H (2025). The association betweend rheumatoid arthritis and stroke risk by serologic status and stroke subtypes. International Journal of Stroke.

[ref-34] Taylor KS, Mahtani KR, Aronson JKJBE-BM (2021). Summarising good practice guidelines for data extraction for systematic reviews and meta-analysis. BMJ Evidence-Based Medicine.

[ref-35] Tiosano S, Yavne Y, Gendelman O, Watad A, Comaneshter D, Shoenfeld Y, Cohen AD, Amital D (2017). Stroke among rheumatoid arthritis patients: does age matter? A real-life study. Neuroepidemiology.

[ref-36] Trommer K, Kostev K, Jacob L, Tanislav C (2021). Increased incidence of stroke and transient ischemic attack in patients with rheumatoid arthritis and ankylosing spondylitis in Germany. Neuroepidemiology.

[ref-37] Ungprasert P, Srivali N, Kittanamongkolchai W (2017). Risk of incident atrial fibrillation in patients with rheumatoid arthritis: a systematic review and meta-analysis. International Journal of Rheumatic Diseases.

[ref-38] Van den Oever IA, Van Sijl AM, Nurmohamed MT (2013). Management of cardiovascular risk in patients with rheumatoid arthritis: evidence and expert opinion. SAGE Journals.

[ref-39] Wells GA, Shea B, O’Connell D, Peterson J, Welch V, Losos M, Tugwell P (2000). The Newcastle-Ottawa Scale (NOS) for assessing the quality of nonrandomised studies in meta-analyses.

[ref-40] Wolfe F, Breundlich B, Straus WL (2003). Increase in cardiovascular and cerebrovascular disease prevalence in rheumatoid arthritis. The Journal of Rheumatology.

[ref-41] Yuan S, Carter P, Mason AM, Yang F, Burgess S, Larsson SC (2022). Genetic liability to rheumatoid arthritis in relation to coronary artery disease and stroke risk. Arthritis & Rheumatology.

[ref-42] Zaman A, Helft G, Worthley S, Badimon J (2000). The role of plaque rupture and thrombosis in coronary artery disease. Atherosclerosis.

[ref-43] Zhang P, Yan XL, Qu Y, Guo ZN, Yang Y (2021). Association between abnormal body weight and stroke outcome: a meta-analysis and systematic review. European Journal of Neurology.

[ref-44] Zhang XW, Yang Z, Li M, Li K, Deng YQ, Tang ZY (2016). Association between dietary protein intake and risk of stroke: a meta-analysis of prospective studies. International Journal of Cardiology.

[ref-45] Zhao W, Wang D, Tan Y, Yang J, Zhang S (2024). Migraine and the correlation between stroke: a systematic review and meta-analysis. Medicine.

[ref-46] Zou K, Xiao FK, Li HY, Zhou Q, Ban L, Yang M, Kuo CF, Zhang W (2017). Risk of cardiovascular disease in Chinese patients with rheumatoid arthritis: a cross-sectional study based on hospital medical records in 10 years. PLOS ONE.

