# Supplementary material for: Rheumatoid arthritis and stroke risk: a systematic review and meta-analysis
Source: PeerJ. 2026 Jan 29;14:e20568. doi: 10.7717/peerj.20568 (PMC12861136; doi:10.7717/peerj.20568)
Supplement: Supplemental Information 3 [file peerj-14-20568-s003.docx]

**Supplementary Material**

**Rheumatoid arthritis and stroke risk: a systematic review and meta-analysis**

**The retrieval strategies and retrieval results of each database are shown in Tables 1-3**.

Table 1: PubMed

| No. | Content | Result |
| --- | --- | --- |
| #1 | Search: "Stroke"[Mesh] Sort by: Most Recent | 194,432 |
| #2 | Search: (((((Stroke*[Title/Abstract]) OR (Cerebrovascular Accident*[Title/Abstract])) OR (Cerebrovascular Apoplexy[Title/Abstract])) OR (Brain Vascular Accident*[Title/Abstract])) OR (Apoplexy[Title/Abstract])) OR (CVAs[Title/Abstract]) Sort by: Most Recent | 381,931 |
| #3 | Search: ("Stroke"[Mesh]) OR ((((((Stroke*[Title/Abstract]) OR (Cerebrovascular Accident*[Title/Abstract])) OR (Cerebrovascular Apoplexy[Title/Abstract])) OR (Brain Vascular Accident*[Title/Abstract])) OR (Apoplexy[Title/Abstract])) OR (CVAs[Title/Abstract])) Sort by: Most Recent | 420,995 |
| #4 | Search: "Arthritis, Rheumatoid"[Mesh] Sort by: Most Recent | 133,529 |
| #5 | Search: Rheumatoid Arthritis[Title/Abstract] Sort by: Most Recent | 133,790 |
| #6 | Search: ("Arthritis, Rheumatoid"[Mesh]) OR (Rheumatoid Arthritis[Title/Abstract]) Sort by: Most Recent | 179,770 |
| #7 | Search: (("Stroke"[Mesh]) OR ((((((Stroke*[Title/Abstract]) OR (Cerebrovascular Accident*[Title/Abstract])) OR (Cerebrovascular Apoplexy[Title/Abstract])) OR (Brain Vascular Accident*[Title/Abstract])) OR (Apoplexy[Title/Abstract])) OR (CVAs[Title/Abstract]))) AND (("Arthritis, Rheumatoid"[Mesh]) OR (Rheumatoid Arthritis[Title/Abstract])) Sort by: Most Recent | 1,102 |
| #8 | Search: ("risk"[Mesh]) OR (risk[Title/Abstract]) Sort by: Most Recent | 3,787,488 |
| #9 | Search: ((("Stroke"[Mesh]) OR ((((((Stroke*[Title/Abstract]) OR (Cerebrovascular Accident*[Title/Abstract])) OR (Cerebrovascular Apoplexy[Title/Abstract])) OR (Brain Vascular Accident*[Title/Abstract])) OR (Apoplexy[Title/Abstract])) OR (CVAs[Title/Abstract]))) AND (("Arthritis, Rheumatoid"[Mesh]) OR (Rheumatoid Arthritis[Title/Abstract]))) AND (("risk"[Mesh]) OR (risk[Title/Abstract])) Sort by: Most Recent | 617 |
|  |  |  |

Table 2 Embase

| No. | Content | Result |
| --- | --- | --- |
| #1 | 'cerebrovascular accident'/exp | 523,226 |
| #2 | 'cerebrovascular accident':ab,ti OR stroke*:ab,ti OR 'cerebrovascular accident*':ab,ti OR 'cerebrovascular apoplexy':ab,ti OR 'brain vascular accident*':ab,ti OR apoplexy:ab,ti OR cvas:ab,ti | 608,101 |
| #3 | #1 OR #2 | 738,234 |
| #4 | 'rheumatoid arthritis'/exp | 287,014 |
| #5 | 'arthritis deformans':ab,ti OR 'arthritis, rheumatoid':ab,ti OR 'arthrosis deformans':ab,ti OR 'beauvais disease':ab,ti OR 'chronic articular rheumatism':ab,ti OR 'chronic polyarthritis':ab,ti OR 'chronic rheumatoid arthritis':ab,ti OR 'disease, beauvais':ab,ti OR 'infantile rheumatoid arthritis':ab,ti OR 'inflammatory arthritis':ab,ti OR 'polyarthritis rheumatica':ab,ti OR 'polyarthritis, primary chronic':ab,ti OR 'primary chronic polyarthritis':ab,ti OR rheumarthritis:ab,ti OR 'rheumatic arthritis':ab,ti OR 'rheumatic polyarthritis':ab,ti OR 'rheumatism, chronic articular':ab,ti OR 'rheumatoid polyarthritis':ab,ti OR 'rheumatoid arthritis':ab,ti | 219,933 |
| #6 | #4 OR #5 | 317,857 |
| #7 | 'risk'/exp | 3,561,137 |
| #8 | risk:ab,ti OR 'risk hypothesis':ab,ti | 4,756,206 |
| #9 | #7 OR #8 | 5,782,370 |
| #10 | #3 AND #6 AND #9 | 2,728 |

Table 3 Cochran Library

| No. | Content | Result |
| --- | --- | --- |
| #1 | MeSH descriptor: [Stroke] explode all trees | 18393 |
| #2 | (Stroke* or Cerebrovascular Accident* or Cerebrovascular Apoplexy or Brain Vascular Accident* or Apoplexy or CVAs):ti,ab,kw | 82246 |
| #3 | #1 or #2 | 82783 |
| #4 | MeSH descriptor: [Arthritis, Rheumatoid] explode all trees | 8188 |
| #5 | (Rheumatoid Arthritis):ti,ab,kw | 19808 |
| #6 | #4 OR #5 | 20234 |
| #7 | MeSH descriptor: [Risk] explode all trees | 55991 |
| #8 | (risk or relative risk or relative risks):ti,ab,kw | 335311 |
| #9 | #7 OR #8 | 339129 |
| #10 | #3 AND #6 AND #9 | 123 |


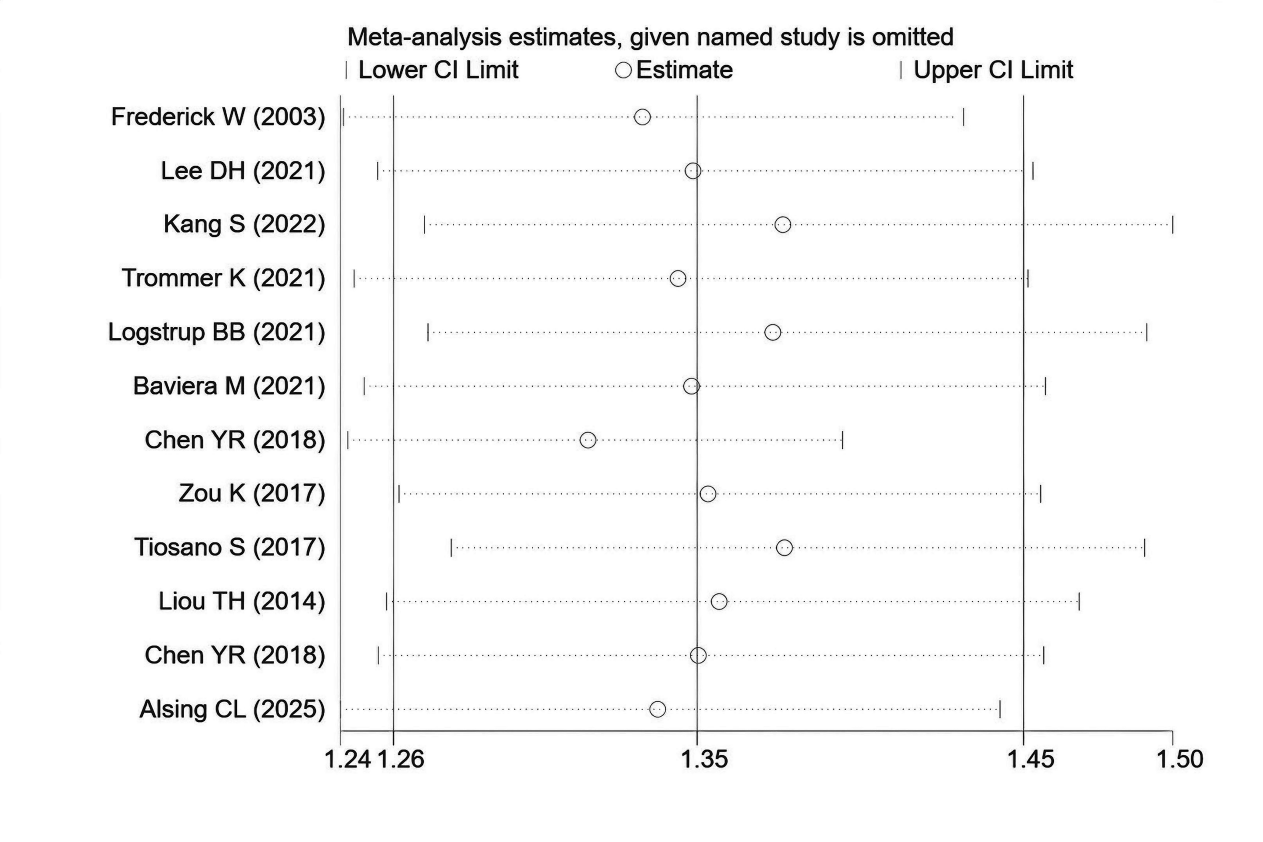


**S1 Fig. Sensitivity analysis** **of rheumatoid arthritis and the risk of stroke.**

**
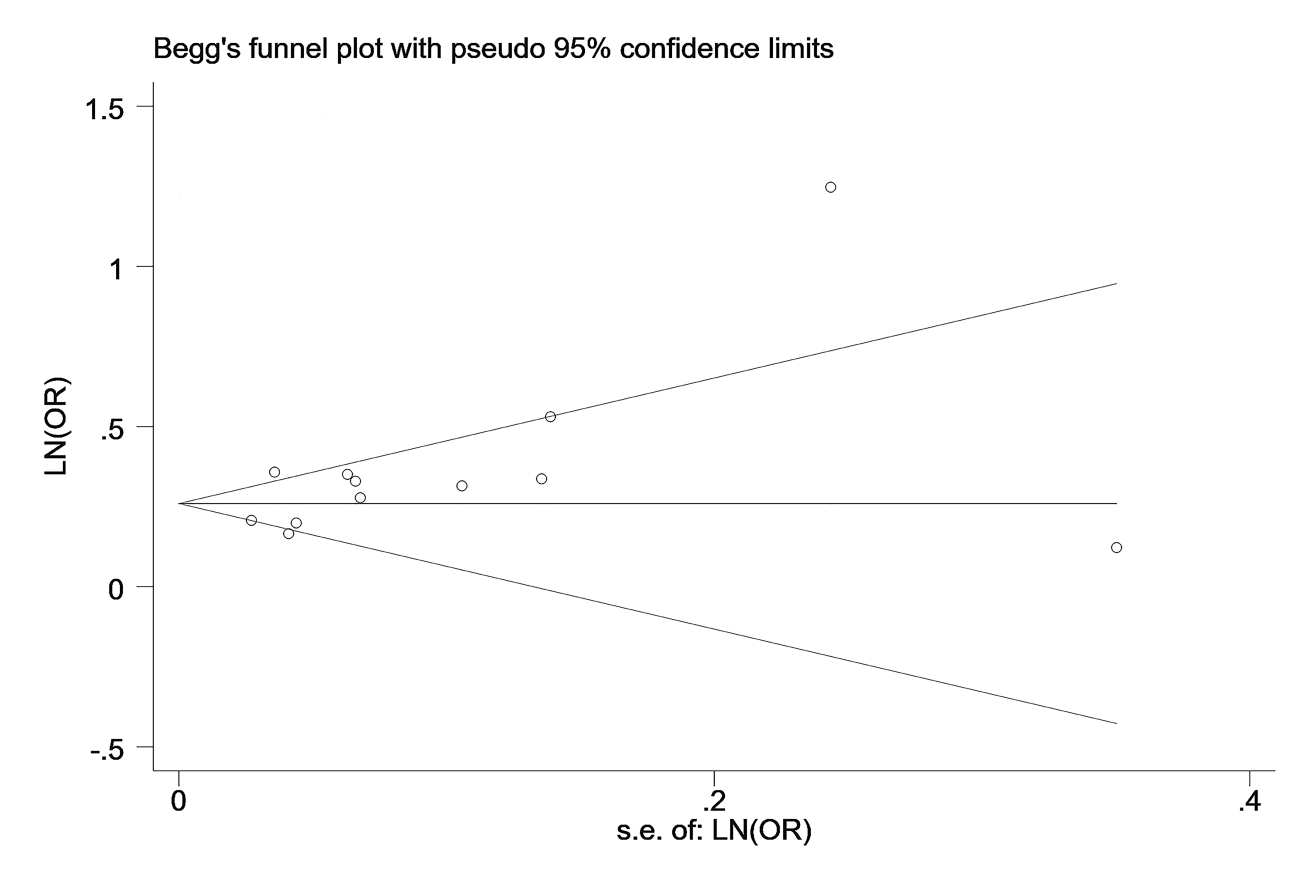
**

**S2 Fig. Begg’s test of the meta-analysis.**

**
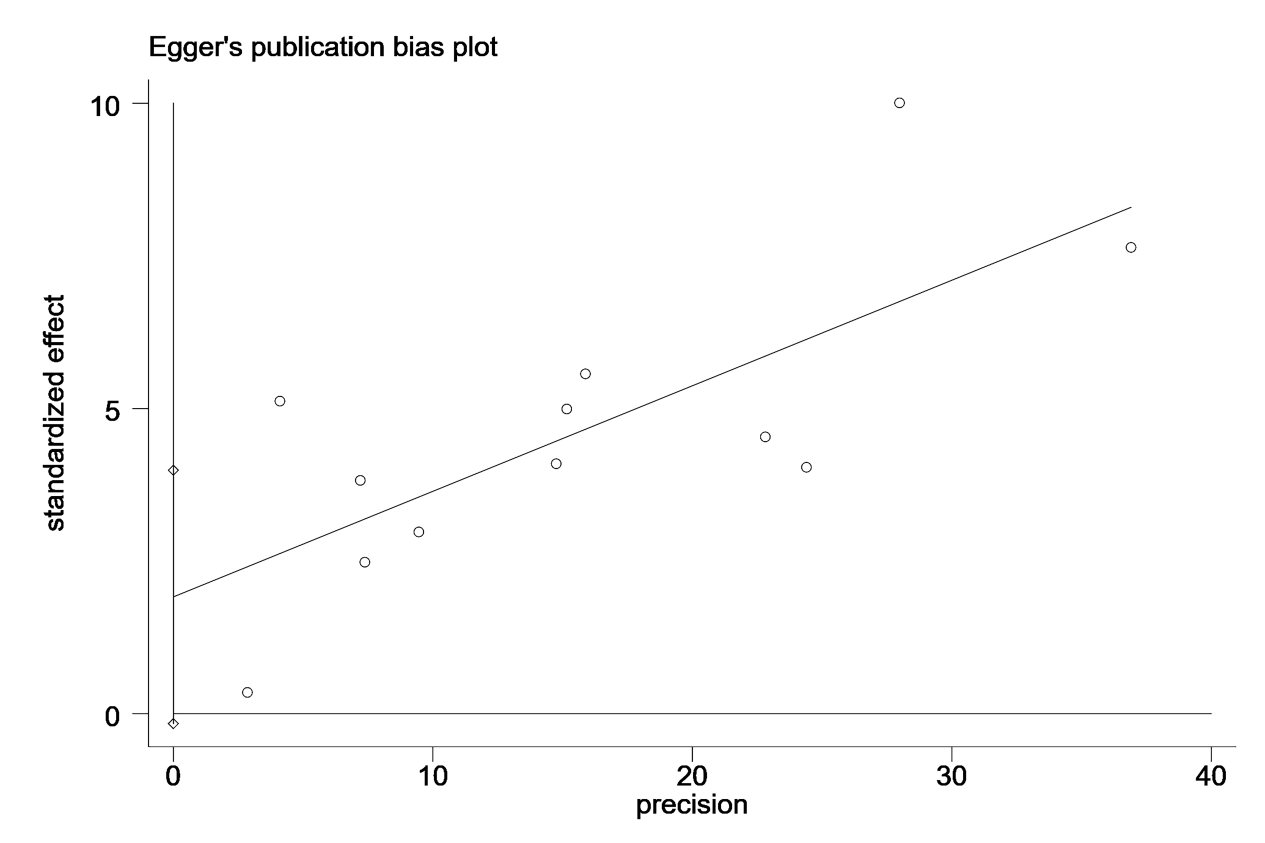
**

**S3 Fig. Egger’s test of the meta-analysis.**

**
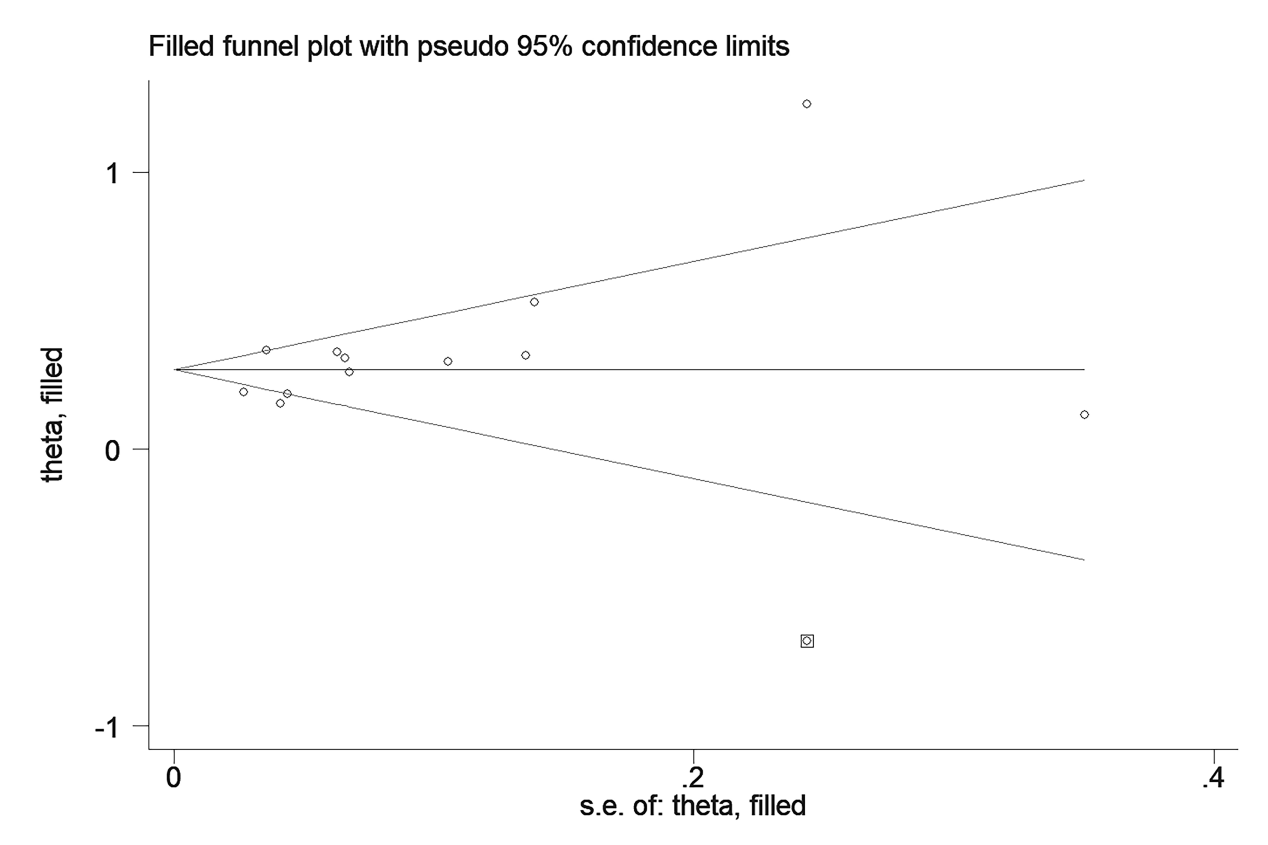
**

**S4 Fig. Funnel plot of Trim and fill method.**
